# Baseline Profile of Participants in the Japan Environment and Children’s Study (JECS)

**DOI:** 10.2188/jea.JE20170018

**Published:** 2018-02-05

**Authors:** Takehiro Michikawa, Hiroshi Nitta, Shoji F. Nakayama, Shin Yamazaki, Tomohiko Isobe, Kenji Tamura, Eiko Suda, Masaji Ono, Junzo Yonemoto, Miyuki Iwai-Shimada, Yayoi Kobayashi, Go Suzuki, Toshihiro Kawamoto, the Japan Environment and Children’s Study Group

**Affiliations:** 1 Japan Environment and Children’s Study Programme Office, National Institute for Environmental Studies, Tsukuba, Japan; 2 Department of Environmental Health, University of Occupational and Environmental Health, Kitakyushu, Japan

**Keywords:** profile, pregnant women, environmental chemicals, birth cohort, Japan

## Abstract

**Background:**

The Japan Environment and Children’s Study (JECS), known as Ecochil-Chosa in Japan, is a nationwide birth cohort study investigating the environmental factors that might affect children’s health and development. We report the baseline profiles of the participating mothers, fathers, and their children.

**Methods:**

Fifteen Regional Centres located throughout Japan were responsible for recruiting women in early pregnancy living in their respective recruitment areas. Self-administered questionnaires and medical records were used to obtain such information as demographic factors, lifestyle, socioeconomic status, environmental exposure, medical history, and delivery information. In the period up to delivery, we collected bio-specimens, including blood, urine, hair, and umbilical cord blood. Fathers were also recruited, when accessible, and asked to fill in a questionnaire and to provide blood samples.

**Results:**

The total number of pregnancies resulting in delivery was 100,778, of which 51,402 (51.0%) involved program participation by male partners. Discounting pregnancies by the same woman, the study included 95,248 unique mothers and 49,189 unique fathers. The 100,778 pregnancies involved a total of 101,779 fetuses and resulted in 100,148 live births. The coverage of children in 2013 (the number of live births registered in JECS divided by the number of all live births within the study areas) was approximately 45%. Nevertheless, the data on the characteristics of the mothers and children we studied showed marked similarity to those obtained from Japan’s 2013 Vital Statistics Survey.

**Conclusions:**

Between 2011 and 2014, we established one of the largest birth cohorts in the world.

## INTRODUCTION

Publicity surrounding diseases caused by environmental pollution, such as Minamata disease (mercury poisoning) and Itai-Itai disease (cadmium poisoning),^1^ ensures that most people know of the detrimental effects on health of highly concentrated chemicals. Japan is not as heavily polluted with such chemicals as it once was, but chemicals are still widely used; discussions now center on the effects of less concentrated chemicals in the environment on human health. The effects of environmental pollution on children’s health, in particular, is of international concern, and the topic has been discussed at the G7/G8 Environment Ministers’ Meeting.^2^ In response, the Japanese Ministry of the Environment proposed a nationwide birth cohort study involving 100,000 mother-child pairs (and fathers, if accessible), and the Japan Environment and Children’s Study (JECS; Ecochil-Chosa in Japanese) was launched in 2011 to evaluate the effects of exposure to chemicals during the fetal stage and in early childhood on children’s health and development; follow-up is planned until the children are 13 years of age.^3^ Several secondary studies using data on approximately 10,000 women who gave birth in 2011 (the first year of recruitment) have already been published in peer-reviewed journals.^4^^–^^10^

Recruitment for the study was closed in March 2014, and the birth data were finalized for processing. This paper summarizes the baseline profiles of all participants (mothers, children, and fathers) at the start of the program.

## METHODS

### Study participants

Details of the JECS concept and design have been published elsewhere.^3^ Briefly, JECS is funded directly by Japan’s Ministry of the Environment and involves collaboration between the Programme Office (National Institute for Environmental Studies), the Medical Support Centre (National Centre for Child Health and Development), and 15 Regional Centres (Hokkaido, Miyagi, Fukushima, Chiba, Kanagawa, Koshin, Toyama, Aichi, Kyoto, Osaka, Hyogo, Tottori, Kochi, Fukuoka, and South Kyushu/Okinawa). Each Regional Centre determined its own study area, consisting of one or more local administrative units (cities, towns or villages) (eTable 1), and was responsible for recruiting women in early pregnancy who resided in its study area. Between January 2011 and March 2014, we contacted pregnant women via cooperating health care providers and/or local government offices issuing Maternal and Child Health Handbooks and registered those willing to participate. The women’s partners (fathers) were also approached, whenever possible, and encouraged to participate. Several Regional Centres later expanded their study areas, because they learned that significant numbers of women residing in adjacent areas gave birth at cooperating health care providers. The Fukushima Centre’s study area was expanded to include the whole of Fukushima Prefecture because of concerns over the effects on health of radioactive fallout from the Fukushima Daiichi Nuclear Power Plant after the March 2011 earthquake and tsunami.

### Assessments during pregnancy and at delivery

#### Questionnaires

Self-administered questionnaires, which were completed by the women during the first trimester and second/third trimester, were used to collect information on demographic factors, medical and obstetric history, physical and mental health, lifestyle, occupation, environmental exposure at home and in the workplace, housing conditions, and socioeconomic status. Most of the questionnaires were distributed to women attending prenatal examinations, but some were sent by post. Completed questionnaires were returned by hand on subsequent prenatal visits or by post. When possible, those who gave incomplete answers were interviewed face-to-face or by telephone. Additionally, the mothers were interviewed about drug use before and during pregnancy.

Between the mothers’ early pregnancy and 1 month after delivery, their male partners were asked to complete a questionnaire covering demographic factors, medical history, physical and mental health, lifestyle, occupation, and environment exposure at home and in the workplace. The survey method was the same as that for the mothers.

#### Medical record transcriptions

Following standard operating procedures, physicians, midwives/nurses, and/or research coordinators transcribed relevant information (medical history, including gravidity and related complications; parity; maternal anthropometry; and infant physical examinations) from medical records.

#### Bio-specimens

Bio-specimens (blood, urine, hair, and umbilical cord blood) were collected during pregnancy and at delivery, and were stored in −80°C freezers, liquid nitrogen tanks, or ordinary-temperature under controlled temperature and humidity. Detailed information about these bio-specimens will be published separately.

### Ethical issues

The Ministry of the Environment’s Institutional Review Board on Epidemiological Studies, and the Ethics Committees of all participating institutions approved the JECS protocol. All participating mothers and fathers had provided written informed consent.

### Statistical analysis

In this paper, we summarized the following characteristics. Maternal profiles, including age, marital status, family composition, and passive smoking (presence of smokers at home), were obtained from the first trimester questionnaire. Information on educational background and household income was collected from the second/third trimester questionnaire. Questions about smoking habits, alcohol consumption (based on the question used in the Japan Public Health Centre-based prospective Study for the Next Generation [JPHC-NEXT]),^11^ and occupation in early pregnancy (based on the 2009 Japan Standard Occupational Classification)^12^ were included in both questionnaires, so data obtained from the first trimester questionnaire was supplemented with data from the second/third trimester questionnaire.^4^ When a participant chose “workers not classifiable by occupation” in the occupation section and then specified an occupation in the comment box, we chose an appropriate job category for that person. Such information as pre-pregnancy height and weight (used for calculating body mass index [BMI] as weight [kg]/height squared [m^2^]), and parity was primarily taken from medical records. When required data were missing from the medical records, questionnaire data were used.

We also summarized profile data on male partners (fathers) via questionnaire: their age and their occupation during their partner’s early pregnancy, along with smoking habits, alcohol consumption, BMI, and educational background (as reported by the female partners). Profile data from medical record on the children were summarized, including delivery information (live birth or not, singleton or multiple birth, gestational age at birth, sex, type of delivery) and anthropometry at birth.

The present study is based on the dataset of jecs-ag-20160424, which was released in June 2016. The birth information in this dataset is not supplemented by any information from the national Vital Statistics Survey. The participants’ profiles were processed in aggregate and also separately for each of the 15 Regional Centres. All analyses were performed with Stata 13 (StataCorp LP, College Station, TX, USA).

## RESULTS

**Figure 1.**
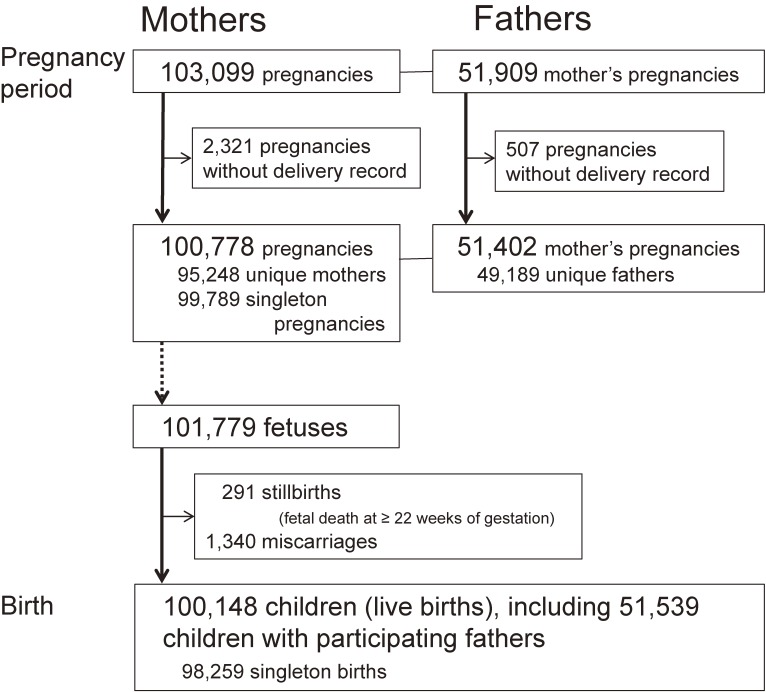
A Japan Environment and Children’s Study cohort flow chart from enrolment to delivery

A JECS cohort flow chart from enrolment to delivery is shown in Figure 1. The study covers a total of 103,099 pregnancies. Excluding the 2,321 pregnancies with no subsequent delivery record, we were left with 100,778 pregnancies resulting in delivery, of which 51,402 (51.0%) involved program participation by male partners. Discounting pregnancies by the same woman, the study involved 95,248 unique mothers and 49,189 unique fathers. The 100,778 pregnancies involved 101,779 fetuses and resulted in 100,148 live births, 291 stillbirths (fetal deaths occurring at ≥22 weeks of gestation), and 1,340 miscarriages. It is difficult to accurately assess the coverage of the children (the number of live births registered in JECS divided by the number of all live births within the study areas) for the entire study period because we recruited women in early pregnancy and later expanded the study areas. In 2013, when recruitment was largely stabilized, however, the child coverage was approximately 45%.

Table 1 shows the response rates of the mothers, fathers, and children for each survey item. The questionnaire and medical record response rates were nearly 100%. The response rates for maternal blood and urine sampling were higher in the second/third trimesters (95.4% for blood and 95.6% for urine) than in the first trimester (88.7% and 88.5%, respectively), mainly because approximately 8% of the pregnancies were registered during the second/third trimesters. Since the first trimester questionnaire was also given to these late participants, its response rate was 98.5%. Although we prioritized the storage of cord blood samples in public cord blood banks, the samples collected from the mothers represented 87.3% of the pregnancies surveyed.

**Table 1.** The response rates of the mothers, fathers, and children for each survey item

	1^st^ trimester		2^nd^/3^rd^ trimester		Birth	
		
	*n*	%	*n*	%	*n*	%
Mother (100,778 pregnancies)						
Questionnaire and interview about drug use	99,300	98.5	97,969	97.2		
Medical record transcription	100,611	99.8			100,778	100
Blood	89,434	88.7	96,098	95.4	94,985	94.3
Urine	89,190	88.5	96,341	95.6		
Hair					X^a^	
Father (51,402 with pregnant partners)						
Questionnaire	50,014	97.3^b^				
Blood	49,661	96.6^b^				
Child (*n* = 101,779)						
Medical record transcription					101,779	100
Cord blood					88,009	87.3^c^
Blood					X^a^	

^a^We have not yet evaluated the data.

^b^Between the mother’s early pregnancy and 1 month after delivery, we distributed a questionnaire to fathers, and collected blood from them.

^c^Response rate of 100,778 pregnancies.

Baseline profiles of the mothers (mean age at delivery, 31.2; standard deviation [SD], 5.1) are shown in Table 2. Most were married (95.6%) and resided with their partner (and their child[ren]) (75.1%). The proportion of those who had received at least 13 years of education was 63.7% for the mothers and 55.8% for the fathers (mother-reported). The distribution of household income peaked at 2 to <4 million Japanese-yen/year (34.6%) and 4 to <6 million yen/year (33.1%). The mothers’ most common occupations in early pregnancy were homemaker (28.8%) and professional/engineering workers (22.3%). Smokers and alcohol drinkers during early pregnancy accounted for 18.2% and 45.9%, respectively. The distribution of baseline profiles did not substantially differ between the total population (about 100,000 mothers) and the sub-population of about 50,000 mothers with male partners participating in the study.

**Table 2.** Baseline profiles of the mothers in the Japan Environment and Children’s Study, 2011–2014

Variables	Total			With male partners participating		
	
	Number of valid response	*n*	(%)	Number of valid response	*n*	(%)
Number of pregnancies	100,778			51,402		
Age at delivery, years	100,768			51,396		
Total, mean (SD)		100,768	31.2 (5.1)		51,396	31.1 (5.0)
<20		893	0.9		374	0.7
20–24		9,229	9.2		4,574	8.9
25–29		27,686	27.5		14,604	28.4
30–34		35,571	35.3		18,387	35.8
35–39		22,713	22.5		11,198	21.8
≥40		4,676	4.6		2,259	4.4
Marital status	98,312			50,624		
Married		94,032	95.6		49,119	97.0
Unmarried		3,444	3.5		1,296	2.6
Divorced/widowed		836	0.9		209	0.4
Family composition	98,123			50,521		
One-person households		653	0.7		216	0.4
A couple only		30,105	30.7		17,386	34.4
A couple with their child(ren)		43,556	44.4		20,769	41.1
A parent with his or her child(ren)		848	0.9		251	0.5
Other households		22,961	23.4		11,899	23.6
Educational background, years	97,004			50,181		
<10		4,704	4.8		2,072	4.1
10–12		30,544	31.5		15,407	30.7
13–16		60,333	62.2		31,902	63.6
≥17		1,423	1.5		800	1.6
Paternal educational background, years	96,387			50,064		
<10		7,049	7.3		2,858	5.7
10–12		35,515	36.8		18,364	36.7
13–16		49,483	51.3		26,405	52.7
≥17		4,340	4.5		2,437	4.9
Household income, million Japanese-yen/year	90,596			47,226		
<2		5,140	5.7		2,300	4.9
2 to <4		31,311	34.6		16,222	34.4
4 to <6		29,942	33.1		15,915	33.7
6 to <8		14,410	15.9		7,668	16.2
8 to <10		5,926	6.5		3,146	6.7
≥10		3,867	4.3		1,975	4.2
Occupation in early pregnancy	97,935			50,506		
Administrative and managerial workers		567	0.6		282	0.6
Professional and engineering workers		21,857	22.3		12,060	23.9
Clerical workers		16,432	16.8		8,569	17.0
Sales workers		5,744	5.9		2,763	5.5
Service workers		15,527	15.9		7,703	15.3
Security workers		242	0.2		138	0.3
Agriculture, forestry and fishery workers		454	0.5		235	0.5
Manufacturing process workers		3,376	3.4		1,889	3.7
Transport and machine operation workers		177	0.2		95	0.2
Construction and mining workers		71	0.1		38	0.1
Carrying, cleaning, packaging, and related workers		678	0.7		290	0.6
Homemaker		28,225	28.8		14,246	28.2
Others (students, inoccupation, workers not classifiable by occupation)		4,585	4.7		2,198	4.4
Smoking habits	99,053			50,897		
Never smoked		57,444	58.0		30,268	59.5
Ex-smokers who quit before pregnancy		23,571	23.8		12,011	23.6
Smokers during early pregnancy		18,038	18.2		8,618	16.9
Passive smoking (presence of smokers at home)^a^	79,910			41,849		
No		66,486	83.2		35,675	85.2
Yes		13,424	16.8		6,174	14.8
Alcohol consumption	99,149			50,937		
Never drank		34,279	34.6		17,666	34.7
Ex-drinkers who quit before pregnancy		19,392	19.6		9,792	19.2
Drinkers during early pregnancy		45,478	45.9		23,479	46.1
Body mass index before pregnancy	100,538			51,358		
<18.5 kg/m^2^		16,272	16.2		8,066	15.7
18.5–24.9 kg/m^2^		73,416	73.0		37,572	73.2
≥25 kg/m^2^		10,850	10.8		5,720	11.1
Parity	100,288			51,212		
0		41,573	41.5		23,280	45.5
1		38,281	38.2		18,555	36.2
≥2		20,434	20.4		9,377	18.3

SD, standard deviation.

^a^Excluding smokers during early pregnancy.

The mean age of the fathers when their partners gave birth was 32.9 (SD, 5.9) years (Table 3); 30.2% were engaged in the professional/engineering works, 47.7% had smoked during their partner’s early pregnancy, and 75.0% had drunk alcohol.

**Table 3.** Baseline profiles of the fathers in the Japan Environment and Children’s Study, 2011–2014

Variables	Number of valid response	*n*	(%)
Number of their partner’s pregnancies	51,402		
Age when their children were born, years	51,104		
Total, mean (SD)		51,104	32.9 (5.9)
<20		198	0.4
20–24		3,173	6.2
25–29		11,659	22.8
30–34		16,867	33.0
35–39		12,738	24.9
≥40		6,469	12.7
Occupation during their partner’s early pregnancy	49,700		
Administrative and managerial workers		2,029	4.1
Professional and engineering workers		15,001	30.2
Clerical workers		4,627	9.3
Sales workers		5,366	10.8
Service workers		5,650	11.4
Security workers		2,065	4.2
Agriculture, forestry and fishery workers		929	1.9
Manufacturing process workers		6,744	13.6
Transport and machine operation workers		2,061	4.1
Construction and mining workers		3,413	6.9
Carrying, cleaning, packaging, and related workers		828	1.7
Homemaker		66	0.1
Others (students, inoccupation, workers not classifiable by occupation)		921	1.9
Smoking habits	49,815		
Never smoked		14,284	28.7
Ex-smokers who quit before their partner’s pregnancy		11,757	23.6
Smokers during their partner’s early pregnancy		23,774	47.7
Alcohol consumption	49,839		
Never drank		10,588	21.2
Ex-drinkers		1,873	3.8
Drinkers		37,378	75.0
Body mass index	49,532		
<18.5 kg/m^2^		1,797	3.6
18.5–24.9 kg/m^2^		34,204	69.1
≥25 kg/m^2^		13,531	27.3

SD, standard deviation.

Table 4 shows baseline profiles of the 100,148 live births. The secondary sex ratio (male/female) was 1.05. Among the 98,259 singleton births, the mean anthropometric values at birth were weight: 3,023 (SD, 420) g, height: 48.9 (SD, 2.3) cm, head circumference: 33.2 (SD, 1.5) cm, and chest circumference: 31.8 (SD, 1.8) cm. The distributions of baseline profiles did not substantially differ between the total population (about 100,000 children) and the sub-population (about 50,000 children with participating fathers).

**Table 4.** Baseline profiles of the children in the Japan Environment and Children’s Study, 2011–2014

Variables	Total			With participating fathers		
	
	Number of valid response	*n*		Number of valid response	*n*	
Number of live births	100,148			51,539		
Singleton births, *n* %		98,259	98.1		50,564	98.1
Gestational age at birth	100,148			51,539		
Total, weeks, mean (SD)		100,148	39.2 (1.7)		51,539	39.2 (1.6)
Preterm births (<37 weeks), *n* %		5,599	5.6		2,644	5.1
Term births (37–41 weeks), *n* %		94,322	94.2		48,763	94.6
Postterm births (≥42 weeks), *n* %		227	0.2		132	0.3
Sex	100,137			51,534		
Male, *n* %		51,316	51.2		26,279	51.0
Female, *n* %		48,821	48.8		25,255	49.0
Type of delivery	99,884			51,413		
Vaginal, *n* %		79,783	79.9		41,212	80.2
Caesarean, *n* %		20,101	20.1		10,201	19.8
Birth weight, g	100,071			51,509		
Total, mean (SD)		100,071	3,008 (434)		51,509	3,015 (425)
Singleton births	98,182			50,534		
Total, mean (SD)		98,182	3,023 (420)		50,534	3,030 (410)
Male, mean (SD)		50,312	3,065 (426)		25,779	3,074 (415)
Female, mean (SD)		47,863	2,979 (408)		24,751	2,984 (399)
Low birth weight, <2,500 g, *n* %		7,981	8.1		3,856	7.6
Birth height, cm	99,785			51,336		
Total, mean (SD)		99,785	48.8 (2.4)		51,336	48.9 (2.3)
Singleton births	97,912			50,368		
Total, mean (SD)		97,912	48.9 (2.3)		50,368	49.0 (2.2)
Male, mean (SD)		50,166	49.2 (2.3)		25,690	49.3 (2.2)
Female, mean (SD)		47,740	48.6 (2.3)		24,675	48.7 (2.2)
Birth head circumference, cm	99,538			51,222		
Total, mean (SD)		99,538	33.2 (1.5)		51,222	33.2 (1.5)
Singleton births	97,692			50,265		
Total, mean (SD)		97,692	33.2 (1.5)		50,265	33.2 (1.5)
Male, mean (SD)		50,054	33.4 (1.5)		25,635	33.4 (1.5)
Female, mean (SD)		47,633	33.0 (1.5)		24,627	33.0 (1.4)
Birth chest circumference, cm	99,489			51,198		
Total, mean (SD)		99,489	31.7 (1.9)		51,198	31.7 (1.8)
Singleton births	97,653			50,245		
Total, mean (SD)		97,653	31.8 (1.8)		50,245	31.8 (1.8)
Male, mean (SD)		50,034	31.9 (1.9)		25,625	31.9 (1.8)
Female, mean (SD)		47,614	31.6 (1.8)		24,617	31.6 (1.8)

SD, standard deviation.

The baseline profiles of the mothers, fathers, and children for each Regional Centre are shown in eTable 2, eTable 3, and eTable 4.

## DISCUSSION

We began registering the participants for the JECS in 2011 and completed registration in 2014, establishing one of the largest birth cohorts in the world. This paper outlines the baseline profiles of the JECS participants.

One strength of this study is that it covers the whole of Japan, from Hokkaido in the north to Okinawa in the south. Although the child coverage was approximately 45% in 2013, the selected characteristics of the mothers and children were comparable with those obtained in the national survey (Table 5).^13^^,^^14^ For example, the proportions of low birth weight (<2,500 g) were 8.2% for JECS in 2013 and 8.3% in the 2013 national Vital Statistics Survey.^13^ The fetal death rate in JECS (3.1 per 1,000 live births and fetal deaths at ≥22 weeks of gestation) was also similar to that in the national survey (3.0).^13^ Therefore, we think we can extrapolate the JECS results to the Japanese general population. Second, the large amount of information collected via questionnaires and/or medical records allows us to investigate the associations between environmental exposure and outcomes after controlling for many covariates, such as lifestyle and physical and social factors. Third, most of the participants provided bio-specimens during pregnancy and at delivery, which will be used to identify new substances in the environment posing health hazards and for gene analyses.

**Table 5.** The selected characteristics of the Japan Environment Children’s Study (JECS) and the national Vital Statistics in 2013

	JECS in 2013	Total population of JECS, 2011–2014	Vital Statistics in 2013^13^
		
	(%)	(%)	(%)
Characteristics of the mothers			
Age at delivery, years			
20–29	36.5	36.6	36.3
30–39	57.8	57.8	57.8
Parity			
0	41.0	41.5	a
Characteristics of the children			
Live births			
Singleton births	98.0	98.1	98.1
Gestational age at birth, weeks			
Term births (37–41 weeks)	94.2	94.2	94.0
Sex			
Male	51.2	51.2	51.2
Female	48.8	48.8	48.8
Type of delivery			
Caesarean	20.3	20.1	19.7^b^
Birth weight, g^c^			
<2,500	8.2	8.1	8.3
2,500 to <3,000	38.5	38.7	39.0
3,000 to <3,500	42.2	42.1	41.8
≥3,500	11.2	11.1	10.9

^a^In Vital Statistics,^13^ birth order has been reported. The proportion of first child among the number of the total births was 46.7% in 2013.

^b^Surveys of Medical Institutions in 2014.^14^

^c^Singleton births only.

Some weaknesses also warrant consideration. First, only about half of the eligible men participated. However, the profiles of the mothers and children did not essentially differ between the total population and the sub-population with paternal participation. Another limitation is that the majority of women were recruited after the latter half of the first trimester. Therefore, we should keep in mind that we did not cover all early miscarriages. In addition, in spite of the large sample size, it is difficult to examine the associations of environmental exposure to chemicals with rare perinatal outcomes, such as amniotic embolism, sudden infant death syndrome, and many individual congenital anomalies.

Information about JECS is available to the public at http://www.env.go.jp/chemi/ceh/. We are following up the participating children by distributing guardian-administered questionnaires every 6 months, starting when the children become 6 months of age, and we are carrying out further chemical analyses of approximately 100,000 blood samples taken from mothers during their second/third trimesters for heavy metals, including lead, cadmium, mercury, manganese, and selenium; these analyses will be completed in 2017. We will soon be able to report on any associations of exposure to heavy metals during pregnancy with pregnancy and reproductive outcomes (eg, preterm delivery, birth weight, and secondary sex ratio).

## APPENDIX A. SUPPLEMENTARY DATA

eTable 1. Study area of each Regional Centre in the Japan Environment and Children’s Study, 2011–2014

eTable 2. Baseline profiles of the mothers according to Regional Centres in the Japan Environment and Children’s Study, 2011–2014

eTable 3. Baseline profiles of the fathers according to Regional Centres in the Japan Environment and Children’s Study, 2011–2014

eTable 4. Baseline profiles of the children according to Regional Centres in the Japan Environment and Children’s Study, 2011–2014

## APPENDIX B.

Members of the Japan Environment and Children’s Study (JECS) as of 2016 (principal investigator, Toshihiro Kawamoto): Hirohisa Saito (National Centre for Child Health and Development, Tokyo, Japan), Reiko Kishi (Hokkaido University, Sapporo, Japan), Nobuo Yaegashi (Tohoku University, Sendai, Japan), Koichi Hashimoto (Fukushima Medical University, Fukushima, Japan), Chisato Mori (Chiba University, Chiba, Japan), Shuichi Ito (Yokohama City University, Yokohama, Japan), Zentaro Yamagata (University of Yamanashi, Chuo, Japan), Hidekuni Inadera (University of Toyama, Toyama, Japan), Michihiro Kamijima (Nagoya City University, Nagoya, Japan), Toshio Heike (Kyoto University, Kyoto, Japan), Hiroyasu Iso (Osaka University, Suita, Japan), Masayuki Shima (Hyogo College of Medicine, Nishinomiya, Japan), Yasuaki Kawai (Tottori University, Yonago, Japan), Narufumi Suganuma (Kochi University, Nankoku, Japan), Koichi Kusuhara (University of Occupational and Environmental Health, Kitakyushu, Japan), and Takahiko Katoh (Kumamoto University, Kumamoto, Japan).
