# Supplementary material for: Baseline Profile of Participants in the Japan Environment and Children’s Study (JECS)
Source: J Epidemiol. 2018 Feb 5;28(2):99–104. doi: 10.2188/jea.JE20170018 (PMC5792233; doi:10.2188/jea.JE20170018)
Supplement: Supplementary file 1 [file je-28-099-s001.pdf]

eTable 1. Study area of each Regional Centre in the Japan Environment and Children's Study, 2011–2014

Regional Centres	Original study area	Additional study area
Hokkaido	Sapporo (Kita-ku and Toyohira-ku), part of Asahikawa, part of Kitami, Oketo, Kunneppu, Tsubetsu and Bihoro	Remaining part of Asahikawa city (from Sep 2011)
Miyagi	Kesennuma, Minamisanriku, Ishinomaki, Onagawa, Osaki, Wakuya, Misato, Kami, Shikama, Kurihara, Tome, Iwanuma, Watari and Yamamoto	
Fukushima	Fukushima, Minamisoma, Namie, Futaba, Okuma, Katsurao, Tomioka, Naraha, Hirono and Kawauchi	Date, Koori, Kunimi, Kawamata (from May 2011) and other areas in Fukushima Prefecture (from Jul 2012)
Chiba	Kamogawa, Minamiboso, Tateyama, Kyonan, Katsuura, Isumi, Onjuku, Otaki, Kisarazu, Sodegaura, Futtsu, Kimitsu and Chiba (Midori-ku)	Ichinomiya (from Dec 2011)
Kanagawa	Yokohama (Kanazawa-ku), Yamato and Odawara	
Koshin	Kofu, Chuo, Koshu, Yamanashi, Fujiyoshida, Ina, Komagane, Tatsuno, Minowa, Iijima, Minamiminowa, Nakagawa and Miyada	
Toyama	Toyama, Kurobe, Asahi and Nyuzen	Uozu (from Dec 2011) and Namerikawa (from Mar 2013)
Aichi	Ichinomiya and Nagoya (Kita-ku)	
Kyoto	Kyoto (Sakyou-ku and Kita-ku), Kizugawa and Nagahama	
Osaka	Kishiwada, Kaizuka, Kumatori, Izumisano, Tajiri, Sennan, Hannan and Misaki	Izumi (from Mar 2013)
Hyogo	Amagasaki	
Tottori	Yonago, Sakaiminato, Daisen, Houki, Nanbu, Kofu, Hino, Nichinan and Hiezu	
Kochi	Kochi, Nankoku, Shimanto and Yusuhara	Konan, Kami, Sukumo, Tosashimizu, Kuroshio, Otsuki and Mihara (from Mar 2012)
Fukuoka	Kitakyushu city (Yahatanishi-ku) and Fukuoka city (Higashi-ku)	
South Kyushu / Okinawa	Minamata, Tsunagi, Ashikita, Amakusa, Reihoku, Kamiamakusa, Hitoyoshi, Nishiki, Asagiri, Taragi, Yunomae, Mizukami, Sagara, Itsuki , Yamae, Kuma, Nobeoka and Miyakojima	

eTable 2. Baseline profiles of the mothers according to Regional Centres in the Japan Environment and Children's Study, 2011–2014

Variables	Hokkaido (%)	Miyagi (%)	Fukushima (%)	Chiba (%)	Kanagawa (%)	Koshin (%)	Toyama (%)	Aichi (%)	Kyoto (%)	Osaka (%)	Hyogo (%)	Tottori (%)	Kochi (%)	Fukuoka (%)	South Kyushu / Okinawa (%)
Number of pregnancies	7,970	9,130	12,882	5,952	6,473	7,175	5,451	5,568	3,920	7,861	5,085	3,026	6,984	7,553	5,748
Age at delivery, years															
Total, mean (SD)	31.4 (4.9)	30.2 (5.1)	30.6 (5.1)	31.1 (5.0)	31.9 (5.1)	31.5 (5.1)	31.8 (4.8)	31.6 (4.9)	32.2 (4.8)	30.8 (5.4)	31.5 (5.0)	31.4 (5.0)	31.3 (5.0)	31.5 (5.0)	30.5 (5.2)
<25	8.6	14.7	12.3	9.7	7.9	8.9	6.4	7.6	5.7	13.4	9.0	8.8	8.6	8.9	12.8
25–29	26.7	31.0	30.2	28.2	24.2	26.2	26.6	25.9	23.3	27.0	25.9	26.8	28.2	25.8	30.4
30–34	37.1	33.2	34.3	34.8	36.0	35.8	36.1	37.4	38.0	32.5	36.0	36.5	36.0	36.6	33.0
≥35	27.6	21.1	23.3	27.3	31.9	29.1	30.9	29.1	33.0	27.1	29.1	27.9	27.2	28.8	23.8
Marital status															
Married	94.8	94.7	95.7	96.5	96.2	95.7	97.5	97.1	97.3	94.4	96.5	96.6	94.7	95.1	95.0
Unmarried	4.4	4.3	3.6	2.9	3.3	3.6	2.1	2.3	2.3	4.5	2.6	2.3	4.1	3.8	3.8
Divorced/widowed	0.9	1.0	0.7	0.7	0.5	0.7	0.4	0.6	0.5	1.1	0.9	1.1	1.2	1.1	1.1
Family composition															
One-person households	1.6	0.4	0.8	0.5	0.3	0.5	0.3	0.4	0.5	0.5	0.4	0.3	1.4	0.8	0.6
A couple only	38.9	19.7	26.8	29.2	36.1	31.3	30.8	34.1	33.3	30.3	38.7	28.3	34.0	33.9	21.8
A couple with their child(ren)	47.4	31.1	37.2	45.8	48.8	41.8	40.7	47.8	47.9	50.3	50.2	40.2	46.9	52.4	47.8
A parent with her child(ren)	1.0	0.7	0.8	0.4	0.6	0.7	0.6	0.8	0.8	1.1	0.8	0.8	1.3	1.2	1.2
Other households	11.0	48.1	34.4	24.2	14.2	25.8	27.7	17.0	17.5	17.8	9.9	30.4	16.5	11.6	28.6
Educational background, years															
<10	4.3	5.5	4.3	4.6	4.4	3.8	3.1	5.2	2.5	7.7	5.8	5.0	4.4	5.8	5.5
10–12	30.4	47.5	39.7	33.4	24.2	27.7	23.2	26.6	16.9	30.5	25.6	32.3	26.6	25.6	42.3
13–16	63.6	46.6	55.4	60.8	68.5	67.2	71.9	66.5	75.7	61.0	66.9	61.4	67.6	66.8	51.7
≥17	1.8	0.4	0.7	1.2	2.9	1.3	1.9	1.7	5.0	0.9	1.7	1.3	1.4	1.9	0.6
Paternal educational background, years															
<10	5.6	8.6	6.2	6.7	6.2	5.4	5.0	7.3	4.6	12.1	8.4	8.1	9.3	7.6	7.4
10–12	31.0	51.7	46.8	39.8	27.1	34.2	30.8	26.9	23.4	37.3	30.7	41.8	33.8	29.9	49.5
13–16	58.1	38.0	44.7	49.2	58.9	56.3	57.3	59.7	60.8	47.8	54.8	45.7	53.7	56.3	41.0
≥17	5.3	1.7	2.3	4.3	7.9	4.0	6.9	6.1	11.2	2.8	6.0	4.5	3.1	6.2	2.1
Household income, million Japanese-yen/year															
<2	5.7	7.4	5.4	3.7	2.6	4.7	2.1	2.8	4.0	6.9	3.7	4.9	9.2	5.4	14.4
2 to <4	37.2	39.9	37.8	32.1	25.0	36.6	25.9	27.5	28.9	37.1	31.8	35.8	36.2	31.9	45.2
4 to <6	32.9	29.4	32.4	35.2	35.6	32.6	36.6	37.3	33.0	33.1	34.8	33.2	30.9	35.1	26.4
6 to <8	14.9	12.6	15.0	17.2	20.3	15.7	21.8	19.3	17.4	14.2	17.6	15.8	16.1	16.4	8.7
8 to <10	5.6	5.9	5.6	7.0	9.7	6.4	8.6	8.4	9.0	6.0	7.9	6.2	5.0	6.9	3.0
≥10	3.8	4.9	3.8	4.9	6.8	4.0	5.1	4.8	7.7	2.8	4.2	4.2	2.7	4.4	2.3
Occupation in early pregnancy															
Administrative and managerial workers	0.4	0.7	0.6	0.5	0.3	0.6	0.5	0.4	0.4	0.7	0.5	0.6	0.9	0.7	0.8
Professional and engineering workers	22.3	19.4	22.6	22.7	19.6	22.0	27.7	17.2	26.6	18.6	18.3	24.7	29.7	22.1	25.2
Clerical workers	14.5	16.1	17.2	14.0	15.5	16.2	21.3	17.8	15.6	14.4	18.6	16.3	19.6	17.2	18.7
Sales workers	5.9	6.5	5.4	5.5	6.2	4.8	4.9	6.1	4.9	6.1	5.8	7.4	6.5	6.4	6.0
Service workers	14.5	18.0	16.7	16.2	14.0	15.4	14.9	12.6	13.5	15.5	14.4	20.5	17.5	13.8	20.6
Security workers	0.3	0.1	0.4	0.3	0.4	0.2	0.3	0.2	0.2	0.3	0.1	0.3	0.3	0.2	0.1
Agriculture, forestry and fishery workers	0.9	0.7	0.4	0.8	0.2	0.6	0.2	0.1	0.2	0.2	0.0	0.4	0.8	0.1	1.3
Manufacturing process workers	1.0	7.9	6.2	1.5	1.7	5.5	5.8	2.0	2.3	2.4	1.5	2.7	1.3	1.3	3.6
Transport and machine operation worke	0.2	0.2	0.2	0.2	0.2	0.1	0.2	0.2	0.1	0.3	0.3	0.2	0.2	0.2	0.1
Construction and mining workers	0.1	0.2	0.1	0.1	0.1	0.1	0.0	0.1	0.0	0.0	0.0	0.0	0.0	0.0	0.1
Carrying, cleaning, packaging, and related workers	0.6	0.8	0.5	0.5	0.9	0.3	0.7	0.7	0.4	1.2	0.6	1.1	0.8	0.9	0.6
Homemaker	34.4	24.6	25.4	33.1	36.4	29.3	20.1	38.1	31.1	34.8	34.9	21.8	18.1	32.3	18.9
Others (students, inoccupation, workers not classifiable by occupation)	5.0	4.9	4.5	4.9	4.6	4.8	3.5	4.7	4.7	5.6	5.0	3.8	4.4	4.9	4.3
Smoking habits															
Never smoked	51.6	50.3	55.6	55.9	60.1	60.1	63.2	65.6	67.2	54.7	61.0	62.3	61.4	58.8	57.7
Ex-smokers who quit before pregnancy	27.8	24.5	24.3	26.4	23.2	24.5	23.8	22.2	22.1	23.3	23.6	23.6	20.1	23.2	22.2
Smokers during early pregnancy	20.6	25.3	20.2	17.7	16.8	15.4	13.0	12.1	10.7	22.0	15.4	14.2	18.5	18.0	20.2
Passive smoking (presence of smokers at home)[a]															
No	78.4	75.7	81.5	85.2	86.9	86.6	86.7	89.2	88.8	80.4	84.5	85.3	82.3	84.5	80.5
Yes	21.6	24.3	18.5	14.8	13.1	13.4	13.3	10.8	11.2	19.6	15.6	14.7	17.7	15.5	19.5
Alcohol consumption															
Never drank	27.3	33.9	33.6	34.8	32.1	36.7	36.4	40.6	37.0	39.6	32.7	40.7	32.5	35.1	32.2
Ex-drinkers who quit before pregnancy	19.1	19.0	18.7	23.1	18.5	20.6	18.7	20.9	19.4	18.9	19.2	20.2	20.3	18.8	20.0
Drinkers during early pregnancy	53.6	47.1	47.8	42.2	49.4	42.6	44.9	38.5	43.7	41.5	48.1	39.1	47.2	46.1	47.9
Body mass index before pregnancy															
<18.5 kg/m^2	17.2	13.9	14.5	14.5	15.4	17.2	17.4	17.0	18.4	17.5	15.1	18.7	15.8	19.3	14.2
18.5–24.9 kg/m^2	72.4	71.7	72.6	73.2	75.0	73.1	75.1	73.8	74.4	72.0	74.9	71.8	74.0	71.7	71.8
≥25 kg/m^2	10.4	14.5	12.8	12.4	9.7	9.7	7.5	9.2	7.2	10.5	10.0	9.6	10.3	9.0	13.9
Parity															
0	45.5	39.7	41.6	39.7	43.9	42.7	41.8	41.5	42.3	40.7	44.3	40.2	43.2	40.6	33.5
1	38.8	37.6	37.5	38.9	39.3	37.9	40.9	40.4	39.9	38.4	38.2	36.8	37.1	38.0	34.2
≥2	15.8	22.7	21.0	21.4	16.9	19.4	17.3	18.1	17.8	20.9	17.6	23.1	19.8	21.4	32.3

SD, standard deviation

[a] Excluding smokers during early pregnancy.

eTable 3. Baseline profiles of the fathers according to Regional Centres in the Japan Environment and Children's Study, 2011–2014

Variables	Hokkaido (%)	Miyagi (%)	Fukushima (%)	Chiba (%)	Kanagawa (%)	Koshin (%)	Toyama (%)	Aichi (%)	Kyoto (%)	Osaka (%)	Hyogo (%)	Tottori (%)	Kochi (%)	Fukuoka (%)	South Kyushu / Okinawa (%)
Number of their partner's pregnancies	2,817	4,143	8,632	3,864	2,418	5,001	3,259	2,547	3,106	2,975	1,868	1,148	2,372	3,784	3,468
Age when their children were born, years															
Total, mean (SD)	33.1 (5.8)	31.6 (6.0)	32.4 (5.9)	33.3 (5.8)	33.6 (5.8)	33.3 (5.8)	33.4 (5.6)	33.6 (5.6)	34.3 (5.7)	32.1 (6.2)	32.8 (5.3)	33.0 (5.8)	32.6 (5.7)	32.9 (5.8)	32.3 (6.0)
<25	5.4	11.1	8.0	5.4	4.8	5.4	4.2	4.1	3.1	10.8	5.4	5.6	5.9	6.3	8.4
25–29	22.8	26.9	24.9	21.8	20.0	21.1	21.5	19.4	16.3	24.6	22.7	22.9	25.7	21.3	26.1
30–34	33.4	32.8	32.7	32.8	31.6	32.7	33.2	35.4	34.2	30.4	35.9	33.4	31.8	34.8	31.9
≥35	38.4	29.2	34.4	40.1	43.6	40.8	41.1	41.1	46.4	34.3	36.1	38.1	36.6	37.7	33.6
Occupation during their partner's early pregnancy															
Administrative and managerial workers	4.5	3.9	4.1	4.1	4.0	4.2	3.4	4.0	5.0	3.9	3.8	2.3	4.0	4.4	4.5
Professional and engineering workers	28.7	23.7	27.6	31.1	35.1	31.0	34.1	33.1	38.4	26.8	32.2	25.8	31.6	30.8	28.8
Clerical workers	11.7	7.9	9.5	8.0	9.9	9.7	9.1	11.4	10.0	7.9	8.9	8.5	9.9	8.8	9.0
Sales workers	14.4	9.3	9.2	8.9	13.3	9.3	9.8	15.6	10.0	11.2	16.2	10.3	11.8	13.2	8.0
Service workers	15.7	11.1	9.8	11.8	11.8	11.5	7.5	9.6	11.0	11.8	12.0	14.7	14.5	10.5	13.6
Security workers	6.9	3.3	4.5	6.3	5.0	3.1	2.7	3.3	3.1	4.1	2.2	9.0	5.0	3.9	2.9
Agriculture, forestry and fishery workers	2.3	3.4	1.7	2.7	0.3	1.7	1.1	0.0	0.8	1.0	0.2	2.6	3.5	0.1	5.9
Manufacturing process workers	3.6	17.5	18.6	12.2	7.3	17.7	19.2	10.3	11.1	14.2	11.2	12.5	6.0	12.1	11.2
Transport and machine operation workers	3.8	5.4	3.7	5.2	4.4	3.0	3.7	4.3	2.8	6.0	3.5	5.8	3.8	4.6	3.9
Construction and mining workers	4.2	10.6	8.0	6.5	4.9	5.8	7.0	5.0	3.8	7.9	6.3	5.6	5.7	7.5	9.0
Carrying, cleaning, packaging, and related workers	0.9	1.8	1.7	1.8	1.1	1.5	1.3	1.9	1.5	2.8	1.9	1.4	1.7	1.8	1.8
Homemaker	0.3	0.1	0.1	0.1	0.1	0.2	0.1	0.1	0.1	0.0	0.2	0.2	0.1	0.2	0.1
Others (students, inoccupation, workers not classifiable by occupation)	3.2	2.1	1.5	1.2	2.9	1.5	1.1	1.4	2.5	2.3	1.5	1.5	2.4	2.1	1.5
Smoking habits															
Never smoked	28.8	19.9	24.8	29.1	33.7	27.6	32.6	39.1	38.7	27.6	36.1	30.4	30.2	28.0	23.0
Ex-smokers who quit before their partner's pregnancy	25.8	18.2	21.5	26.3	25.1	25.1	24.7	26.7	26.6	23.7	24.2	26.7	22.8	22.9	21.6
Smokers during their partner's early pregnancy	45.5	61.9	53.7	44.5	41.2	47.3	42.7	34.3	34.7	48.7	39.8	42.9	47.0	49.1	55.4
Alcohol consumption															
Never drank	19.4	20.1	19.6	20.1	22.3	21.9	21.0	26.8	21.5	24.8	22.8	24.2	22.2	22.9	16.4
Ex-drinkers	4.1	4.0	3.7	4.0	3.7	3.8	3.4	4.3	3.9	4.2	3.0	3.9	4.6	3.4	2.9
Drinkers	76.5	75.9	76.7	75.9	74.0	74.4	75.6	68.9	74.6	71.0	74.3	71.9	73.2	73.7	80.7
Body mass index before pregnancy															
<18.5 kg/m^2	3.7	3.7	3.5	2.6	4.1	3.6	3.4	4.2	3.6	3.1	4.2	5.0	4.1	4.1	3.3
18.5–24.9 kg/m^2	67.9	64.9	67.6	67.1	71.1	71.0	74.5	71.6	73.8	69.0	71.5	71.7	68.6	68.4	64.6
≥25 kg/m^2	28.4	31.4	28.9	30.3	24.8	25.4	22.1	24.3	22.5	27.9	24.3	23.4	27.3	27.6	32.2

SD, standard deviation

eTable 4. Baseline profiles of the children according to Regional Centres in the Japan Environment and Children's Study, 2011–2014

Variables	Hokkaido	Miyagi	Fukushima	Chiba	Kanagawa	Koshin	Toyama	Aichi	Kyoto	Osaka	Hyogo	Tottori	Kochi	Fukuoka	South Kyushu / Okinawa
Number of live births	7904	9053	12857	5942	6393	7162	5392	5535	3901	7838	5044	3033	6883	7519	5692
Singleton births, %	98.2	98.3	98.1	97.9	98.6	98.2	98.5	98.2	97.7	97.5	98.2	97.5	98.5	97.7	98.3
Gestational age at birth															
Total, weeks, mean (SD)	39.1 (1.7)	39.1 (1.7)	39.3 (1.6)	39.0 (1.7)	39.2 (1.6)	39.1 (1.6)	39.3 (1.6)	39.2 (1.7)	39.2 (1.6)	39.2 (1.7)	39.2 (1.7)	39.3 (1.6)	39.1 (1.7)	39.2 (1.7)	39.1 (1.6)
Preterm births (<37 weeks), %	6.1	6.0	5.1	6.2	5.4	5.4	4.9	5.8	5.5	5.8	5.3	4.4	5.9	5.6	5.9
Term births (37–41 weeks), %	93.8	93.9	94.7	93.6	94.5	94.5	94.6	93.8	94.4	93.8	94.3	95.1	93.9	94.0	94.0
Postterm births (≥42 weeks), %	0.1	0.1	0.1	0.2	0.1	0.2	0.5	0.4	0.1	0.4	0.4	0.5	0.1	0.4	0.2
Sex															
Male, %	51.1	51.2	51.6	51.4	52.4	50.3	51.2	51.2	51.2	51.4	52.4	49.5	50.8	50.9	51.7
Female, %	48.9	48.8	48.5	48.7	47.6	49.8	48.8	48.8	48.8	48.6	47.6	50.5	49.2	49.1	48.3
Type of delivery															
Vaginal, %	78.6	81.4	79.3	78.3	84.7	81.5	82.8	80.5	79.2	79.5	78.7	80.6	74.0	81.4	78.0
Caesarean, %	21.4	18.6	20.7	21.7	15.3	18.5	17.3	19.6	20.8	20.5	21.3	19.4	26.0	18.6	22.1
Birth weight, g															
Total, mean (SD)	3,019 (439)	3,039 (450)	3,013 (427)	2,998 (434)	2,998 (418)	2,975 (426)	3,014 (422)	3,021 (438)	3,018 (420)	3,001 (433)	3,016 (440)	2,994 (425)	2,979 (425)	2,994 (445)	3,043 (449)
Singleton births															
Total, mean (SD)	3,034 (423)	3,053 (436)	3,027 (415)	3,015 (414)	3,008 (408)	2,988 (412)	3,025 (412)	3,035 (426)	3,035 (404)	3,019 (418)	3,032 (423)	3,013 (405)	2,992 (411)	3,013 (425)	3,054 (441)
Low birth weight (<2,500 g), %	7.7	7.7	7.8	8.5	8.6	9.2	7.5	7.9	7.4	8.2	7.6	8.7	8.9	8.6	7.6
Birth height, cm															
Total, mean (SD)	48.7 (2.5)	49.3 (2.5)	49.0 (2.4)	48.4 (2.4)	49.0 (2.2)	49.1 (2.3)	48.6 (2.2)	49.6 (2.3)	48.7 (2.3)	48.2 (2.3)	48.7 (2.4)	48.2 (2.4)	48.5 (2.4)	49.1 (2.6)	48.8 (2.3)
Singleton births, mean (SD)	48.8 (2.4)	49.3 (2.4)	49.0 (2.3)	48.5 (2.3)	49.1 (2.1)	49.2 (2.3)	48.7 (2.2)	49.7 (2.3)	48.8 (2.2)	48.3 (2.2)	48.8 (2.3)	48.3 (2.3)	48.6 (2.3)	49.2 (2.4)	48.8 (2.3)
Birth head circumference, cm															
Total, mean (SD)	33.3 (1.5)	33.1 (1.6)	33.2 (1.5)	33.1 (1.5)	33.3 (1.5)	33.1 (1.6)	33.1 (1.5)	33.0 (1.5)	33.2 (1.5)	33.2 (1.5)	33.3 (1.5)	33.1 (1.4)	33.1 (1.5)	33.1 (1.6)	33.1 (1.5)
Singleton births, mean (SD)	33.4 (1.5)	33.2 (1.6)	33.2 (1.5)	33.1 (1.5)	33.3 (1.5)	33.1 (1.5)	33.1 (1.5)	33.0 (1.5)	33.2 (1.5)	33.2 (1.5)	33.3 (1.5)	33.1 (1.4)	33.1 (1.5)	33.1 (1.5)	33.1 (1.5)
Birth chest circumference, g															
Total, mean (SD)	31.7 (1.9)	31.6 (1.9)	31.7 (1.9)	31.6 (1.9)	31.8 (1.9)	31.7 (1.9)	31.8 (1.9)	31.8 (1.9)	31.7 (1.9)	31.7 (1.9)	31.8 (1.9)	31.7 (1.8)	31.6 (1.9)	31.7 (1.9)	31.9 (1.9)
Singleton births, mean (SD)	31.8 (1.9)	31.7 (1.9)	31.7 (1.8)	31.7 (1.8)	31.9 (1.8)	31.7 (1.8)	31.8 (1.8)	31.9 (1.9)	31.7 (1.8)	31.8 (1.9)	31.9 (1.8)	31.8 (1.7)	31.7 (1.8)	31.7 (1.8)	32.0 (1.8)

SD, standard deviation
